# Geospatial Access to Emergency Obstetric Surgery in Indonesia: Is Travel Time for Access Too Long?

**DOI:** 10.5334/aogh.4598

**Published:** 2024-12-28

**Authors:** Brahmana Askandar Tjokroprawiro, Hanif Ardiansyah Sulistya, Farizal Rizky Muharram, Renata Alya Ulhaq, Alifina Izza, Budi Prasetyo, Khoirunnisa Novitasari, Budi Wiweko, Pandu Hanindito Habibie, Arya Ananda Indrajaya Lukmana, Muhammad Muhibuddin Hilmy Asari

**Affiliations:** 1 Department of Obstetrics and Gynecology, Faculty of Medicine, Universitas Airlangga/Dr. Soetomo General Academic Hospital, Surabaya, Indonesia; 2 Indonesian Society of Obstetricians and Gynecologists, Jakarta, Indonesia; 3 ARC Institute, Surabaya, Indonesia; 4 Department of Global Health and Social Medicine, Harvard Medical School, Boston, MA, USA; 5 Department of Obstetrics and Gynecology, Faculty of Medicine, Universitas Indonesia/Dr. Cipto Mangunkusumo General Hospital, Jakarta, Indonesia; 6 Indonesia Medical Education Research Institute (IMERI), Faculty of Medicine Universitas Indonesia – Jakarta, Indonesia; 7 Faculty of Medicine, Universitas Airlangga, Dr. Soetomo General Academic Hospital, Surabaya, Indonesia

**Keywords:** Geospatial analysis, Emergency obstetric surgical care, Global surgery, Indonesia

## Abstract

*Background:* Ensuring timely access to safe and affordable surgery within a travel time of a 2‑h drive or 30‑min walk is crucial for achieving universal health coverage, as endorsed by the Lancet Commission on Global Surgery (LCoGS). In this study, we aimed to quantify the percentage of Indonesian women of reproductive age (WRA) who can access a hospital with emergency obstetric and gynecological services within this time frame. In addition, we aimed to identify the underserved populations.

*Methods:* We identified hospitals across 38 provinces using the database from the Indonesian Society of Obstetricians and Gynecologists (ISOG) and the Indonesian Ministry of Health database that provide emergency obstetric services. We conducted geospatial analysis using the cost of distance and service area tools in ArcGIS Pro with WRA population data derived from Facebook’s high‑resolution settlement layer (HRSL) maps.

*Results:* Of the 3,202 recorded hospitals, 2,855 (89.2%) had an obstetric gynecologist (OBGYN). The workforce of 5,305 OBGYNs consisted of 4,857 (91.6%) actively practicing OBGYNs, of which 3,405 (64.2%) practice in hospitals only. Of the WRA population, 94.5% lived within 2 h of a facility. However, eight provinces had low timely access to these hospitals.

*Conclusion:* Indonesia provides universal health coverage; however, stark disparities exist in the geographic access to emergency obstetric surgical care in certain provinces. Geospatial mapping and survey data work together to aid in assessing the strength of the surgical system and in identifying gaps in geographic access to timely surgery.

## Background

In 2015, the Lancet Commission on Global Surgery (LCoGS) proposed six core surgical indicators to monitor access to safe and affordable surgical and anesthesia care [1]. The indicators were developed to define, assess, and inform the surgical system on preparedness, service delivery, and cost‑efficiency. The first indicator measures the proportion of a country’s population living within 2 h of a bellwether‑capable facility, which provides cesarean section, laparotomy, and management of open fractures [2]. This metric serves as a proxy for timely access to essential surgery, with an 80% 2‑h access (2HA) rate considered adequate [3].

Geospatial mapping of 2HA has been conducted in various regions worldwide; however, comprehensive studies in Indonesia are lacking. Indonesia, an archipelagic nation comprising approximately 17,000 islands, presents unique geographical challenges for healthcare delivery [4]. As the world’s fourth most populated country, with a population exceeding 273 million in 2020, the dispersion of its population across numerous islands hinders the provision of timely healthcare services. Previous studies have highlighted significant barriers to timely, safe, and affordable surgery in Southeast Asia, underscoring the need for targeted research in this context [5, 6].

In this study, we conducted a geospatial analysis of access to emergency obstetric services within a 2‑h drive or 30‑min walk in Indonesia, focusing on the country’s substantial geographical barriers. There are two main objectives in this study: first, to determine the proportion of the reproductive‑age population in Indonesia that can reach a hospital with emergency obstetric services within a specified timeframe and, second, to identify areas lacking adequate access and suggest potential sites for infrastructure improvements. The purpose is to provide a comprehensive estimate of access based on population distribution, hospital locations, and road networks, which can assist the Indonesian government and other stakeholders in making informed national decisions.

## Methods

In this study, we adopted an observational cross‑sectional design to evaluate geospatial access to emergency obstetric surgery services in Indonesia. Secondary data sources included the hospital location from the Ministry of Health (MoH), obstetric gynecologist (OBGYN) practice location from the electronic management office (EMOP) of the Indonesian Society of Obstetrics and Gynecology (ISOG/POGI), and population estimates from the Facebook high‑resolution settlement layer (HRSL). We focused on women of reproductive age (15–49 years) and evaluated their access to hospitals with obstetric services within a 2‑h drive or 30‑min walk. The analysis identified underserved areas by mapping population density and hospital distribution and aimed to inform surgical workforce planning and infrastructure development in Indonesia. The latest maternal mortality ratio (MMR) data available for 2020 were obtained from www.bps.go.id [7].

### Hospital and obstetric gynecologist data

Indonesia can be divided into seven main island groups: Sumatra, Java, Bali and Lesser Sunda, Kalimantan, Sulawesi, the Maluku Islands, and Papua. These islands are divided into 38 provinces distributed across three time zones; of these provinces, five are Special Autonomy Provinces (Aceh, Yogyakarta, Jakarta, Papua, and West Papua).

In December 2023, all Indonesian hospitals were identified using a comprehensive database of accredited hospitals published by the MoH [8]. According to the Indonesian MoH Decree No. 56 of 2014, all hospitals are classified into two types using the type of service provided. General hospitals provide healthcare services across all fields and for all disease types. In contrast, specialty hospitals primarily focus on a specific field or disease type based on discipline, age group, organ, type of disease, and other specializations. Hospitals are classified into public and private using their management system. Public hospitals are managed by the state or local government or a non‑profit legal entity. In addition, private hospitals are managed by legal entities with the purpose of profiting from the private organizations or legal entities.

For this study, all 3,202 recorded hospitals from the Indonesian MoH database were retrieved on 4 September 2024 and matched with the database of OBGYN, including their practice locations, from ISOG. Following manual data cleaning, a final list of 2,566 hospitals was obtained. Subsequently, the Global Positioning System (GPS) coordinates of each hospital were identified using Google Earth Pro, which automatically tabulated the latitude and longitude of hospitals using their address. Hospital names and their GPS coordinates were input into ArcGIS Pro (version 3.2) and stored as point features.

### Population data

The spatial population data for women of reproductive age (WRA), defined as those aged 15–49 years, were obtained from Facebook HRSL for 2020, the most recent available year [9]. The detailed methodology for these data, combining census data, satellite imagery, and machine learning algorithms, was explained in another study [10].

### Network analysis to measure geospatial access to timely obstetric surgical care

The LCoGS outlined timely access to surgical care as 2‑h access to a hospital providing surgical care. In addition, 30‑min access was measured to evaluate access to essential obstetric care, as recommended by the American College of Obstetricians and Gynecologists (ACOG). This suggests a 30‑minute benchmark for access to emergency cesarean sections (CS).

We utilized the Network Analyst tool from ArcGIS Pro with a road network database sourced from the Indonesian Geospatial Information Agency (Badan Informasi Geospatial Indonesia), scaled at 1:250,000, to estimate walking and driving time. The walking speed was estimated to be 5 km/h; in contrast, the vehicle driving speed was set to 50 km/h. Service area maps were generated around each hospital with available OBGYNs, delineating areas reachable within 30 min of walking and 2 h of driving with a vehicle. Speed limits were embedded within the road network dataset, with the assumption that all patients always adhered to these speed limits.

First, a service‑area analysis layer was created by importing a road network map. Subsequently, driving time parameters and hospital locations were selected, and a solving tool was used to perform the analysis. The population estimate raster was combined with the driving time analysis areas to calculate the total population for each travel time. These data were summarized using zonal statistics. The detailed methodology for geographic information system (GIS) analysis was previously described in another study [11].

### Ethical approval

This study was approved by the Health Research Ethics Committee of the Faculty of Public Health, Universitas Airlangga (No. 62/EA/KEPK/2024).

## Results

**Figure 1:**
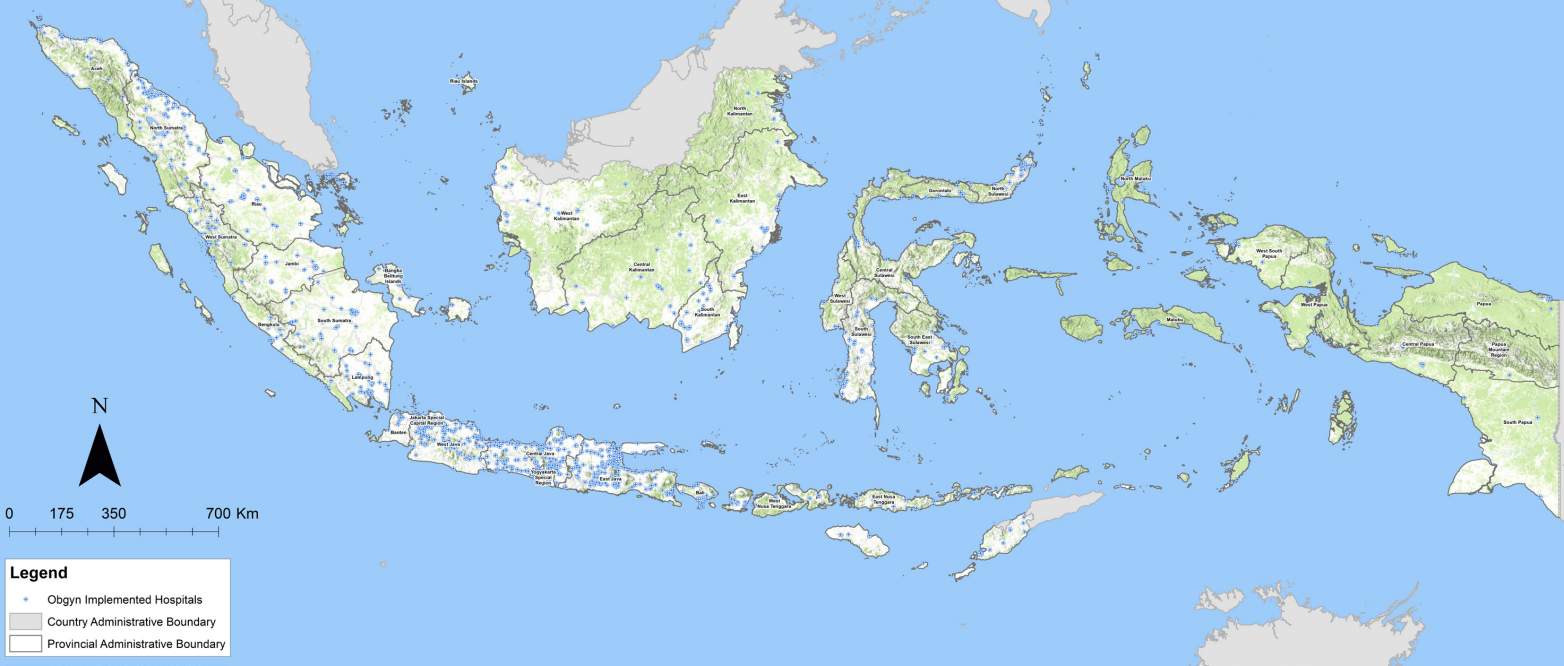
Location of hospitals with available obstetric gynecologists within 38 provinces in Indonesia.

**Figure 2:**
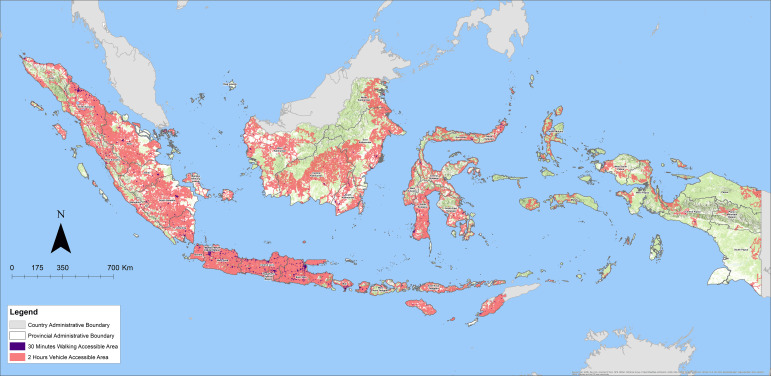
30‑minute walking and 2HA to hospitals with available obstetric gynecologists within 37 provinces.

There are 2,855 hospitals across Indonesia with an available obstetric gynecologist (OBGYN) providing emergency obstetric surgical services (Figure 1). In Indonesia, 89.2% of 3,202 hospitals have an obstetrician‑gynecologist who can provide emergency obstetric surgical services. Overall, 94.5% of the population lives within 2 h of a hospital that provides emergency obstetric surgery, which is notably above the LCoGS target rate of 80% for every country by 2030. Among the seven island groups, five met the LCoGS indicator, ensuring that at least 80% of the population reached a hospital capable of performing emergency CS within 2 h of travel time. The total WRA population was highest in the Java Island group, with 99.2% having 2‑h access to emergency obstetric surgical care (EOSC). In contrast, the Maluku Islands and Papua had the lowest WRA populations and the lowest 2‑h access coverage at 69.2% and 60.7%, respectively (Figure 2; Table 1).

**Table 1:** The 2HA to the hospital with available obstetric‑gynecologist based on island groups.

ISLAND GROUP	TOTAL WRA POPULATION	TOTAL HOSPITALS, *N*	APO, *N* (%)	APO‑TO‑10,000‑WRA RATIO	HAPO, *N* (%)	WRA POPULATION WITHIN 2HA, *N* (%)
Sumatra	15,976,586	755	1,023 (21.06)	0.64	671 (88.87)	14,697,188 (91.99)
Java	41,832,819	1,614	2,766 (56.95)	0.66	1,509 (93.49)	41,508,923 (99.23)
Bali and Lesser Sunda	4,137,334	191	318 (6.55)	0.77	160 (83.77)	3,897,049 (94.19)
Kalimantan	4,716,806	219	289 (5.95)	0.61	182 (83.11)	3,929,182 (83.30)
Sulawesi	5,552,861	297	362 (7.45)	0.65	247 (83.16)	5,153,142 (92.80)
Maluku Islands	919,318	53	36 (0.74)	0.39	38 (71.70)	636,478 (69.23)
Papua	2,081,918	73	63 (1.30)	0.30	48 (65.75)	1,263,995 (60.71)
**INDONESIA**	**75,217,642**	**3,202**	**4,857**	**0.65**	**2,855 (89.2)**	**71,085,957 (94.51)**

WRA = women of reproductive age.

APO = actively practicing OBGYN.

OBGYN = obstetric gynecologist.

HAPO = hospital with an actively practicing OBGYN.

### Obstetric gynecologist workforce and hospital characteristics

Of 5,305 OBGYNs in Indonesia, 448 (8.4%) were either inactive or retired. The provinces with the highest percentages of inactive or retired OBGYNs were the Riau Islands (29.8%) and Riau (21.2%), indicating significant regional disparities in active OBGYN practitioners. In addition, 120 OBGYNs (2.3%) worked exclusively in private clinics, with the highest proportions in the Bangka Belitung Islands (6.5%) and East Kalimantan (5.0%) (Supplementary Table 1).

Meanwhile, 189 and 20 general and surgical hospitals (7.0% and 64.5%), respectively, across various provinces did not have actively practicing OBGYNs (APO). Notably, in regions such as Bengkulu, North Maluku, and Southeast Sulawesi, all surgical hospitals lacked OBGYNs. In addition, other hospital categories consistently demonstrated the highest proportion without OBGYNs, reaching 85.6% nationally (Supplementary Table 2).

Furthermore, 108 Class D hospitals (12.3%) and 57 Class D primary hospitals (82.6%) lacked actively practicing OBGYNs, indicating a significant gap in specialist availability among lower‑classified hospitals. Notably, class C hospitals were affected, with 109 facilities (6.3%) lacking OBGYNs, which reflects the disparities in the availability of OBGYNs across various hospital classes, particularly in rural or underserved areas (Supplementary Figure 1; Supplementary Table 3).

### A 2‑h access analysis by province

At the provincial level, the geospatial analysis showed that eight provinces did not achieve the first LCoGS indicator target of 80%, including West Kalimantan, the Riau Islands, Maluku, North Maluku, Papua, Papua Mountains, South Papua, and Central Papua. Access to EOSC is lowest (42.8%) in South Papua. In contrast, 2HA to EOSC is highest in Jakarta, which is the current capital of Indonesia, with 100% and 95% of the WRA population within 2HA and 30‑min walking time, respectively.

The 2HA to EOSC is largely affected by population distribution compared with the island distribution and land area. The densely populated areas had higher access rates. For example, Jakarta has a 2HA of 100% and the highest population density at 4,849 WRA per km^2^. Conversely, access rates were lowest in South Papua (42.8%) and the Papua Mountains (46.7%), with WRA population densities of 2 and 5 per km^2^, respectively (Table 2).

**Table 2:** Geographic access of women of reproductive age (WRA) in Indonesia to hospital with available obstetric gynecologist by provinces.

PROVINCE	DISTRICT WITHOUT HAPO, *N* (%)	TOTAL HOSPITALS, *N*	HAPO, *N* (%)	APO, *N* (%)	WRA POPULATION IN 2020, *N*	APO‑TO‑10,000‑WRA RATIO	WRA POPULATION WITHIN 30 MIN WALKING TIME, *N* (%)	WRA POPULATION WITHIN 2‑H DRIVING TIME, *N* (%)
Aceh	–	80	69 (86.3)	105	1,306,348	0.80	209,520 (16.0)	1,210,915 (92.7)
Bali	–	81	71 (87.7)	193	1,308,095	1.48	501,731 (38.4)	1,253,068 (95.8)
Banten	–	129	124 (96.1)	242	3,998,283	0.61	1,582,327 (39.6)	3,979,666 (99.5)
Bengkulu	–	26	24 (92.3)	32	503,626	0.64	95,544 (19.0)	469,932 (93.3)
Jakarta	–	190	163 (85.8)	799	3,219,955	2.48	3,070,072 (95.4)	3,219,955 (100)
Yogyakarta	–	81	68 (84.0)	98	1,018,902	0.96	437,352 (42.9)	1,016,642 (99.8)
Gorontalo	–	20	18 (90.0)	22	340,528	0.65	100,152 (29.4)	332,417 (97.6)
Jambi	1 (10.0)	42	38 (90.5)	56	1,078,450	0.52	250,551 (23.2)	943,756 (87.5)
West Java	–	425	407 (95.8)	553	14,018,250	0.39	5,686,588 (40.6)	13,908,896 (99.2)
Central Java	–	355	334 (94.1)	490	8,771,855	0.56	1,948,897 (22.2)	8,737,013 (99.6)
East Java	–	434	413 (95.2)	584	10,805,574	0.54	2,931,042 (27.1)	10,646,751 (98.5)
West Kalimantan	–	58	48 (82.8)	67	1,363,613	0.49	185,373 (13.6)	1,090,023 (79.9)
South Kalimantan	–	51	48 (94.1)	73	1,237,110	0.59	314,888 (25.5)	1,027,353 (83.0)
Central Kalimantan	–	33	27 (81.8)	39	737,262	0.53	75,906 (10.3)	607,991 (82.5)
East Kalimantan	1 (10.0)	60	49 (81.7)	94	1,162,998	0.81	347,169 (29.9)	1,027,382 (88.3)
North Kalimantan	–	17	10 (58.8)	16	215,823	0.74	62,988 (29.2)	176,433 (81.8)
Bangka Belitung Islands	–	28	25 (89.3)	31	432,397	0.72	94,105 (21.8)	370,031 (85.6)
Riau Islands	1 (14.3)	36	32 (88.9)	40	849,664	0.47	309,755 (36.5)	649,996 (76.5)
Lampung	–	82	78 (95.1)	96	2,297,241	0.42	394,261 (17.2)	2,145,035 (93.4)
Maluku	2 (18.2)	30	20 (66.7)	22	518,999	0.42	89,251 (17.2)	348,281 (67.1)
North Maluku	1 (10.0)	23	18 (78.3)	14	400,319	0.35	62,055 (15.5)	288,197 (72.0)
West Nusa Tenggara	–	46	42 (91.3)	64	1,454,108	0.44	290,755 (20.0)	1,399,956 (96.3)
East Nusa Tenggara	1 (4.5)	64	47 (73.4)	61	1,375,131	0.44	221,641 (16.1)	1,244,025 (90.5)
Papua	3 (33.3)	18	14 (77.8)	21	252,945	0.83	91,311 (36.1)	169,889 (67.2)
Highland Papua	6 (75.0)	9	2 (22.0)	5	590,414	0.08	28,796 (4.9)	275,836 (46.7)
South Papua	–	8	7 (87.5)	6	207,265	0.29	21,697 (10.5)	88,804 (42.9)
Central Papua	4 (50.0)	14	6 (42.9)	8	719,576	0.11	109,527 (15.2)	475,497 (66.1)
Riau	1 (8.3)	72	72 (87.8)	115	2,214,141	0.52	394,501 (17.8)	1,954,283 (88.3)
West Sulawesi	1 (16.7)	16	10 (62.5)	10	414,829	0.24	37,478 (9.0)	392,633 (94.7)
South Sulawesi	1 (4.2)	124	106 (85.5)	177	2,463,008	0.72	651,761 (26.5)	2,350,348 (95.4)
Central Sulawesi	–	40	31 (77.5)	39	844,287	0.46	114,184 (13.5)	746,980 (88.5)
Southeast Sulawesi	3 (17.6)	39	34 (87.2)	37	778,121	0.48	130,629 (16.8)	668,822 (86.0)
North Sulawesi	1 (6.7)	58	48 (82.8)	77	712,088	1.08	225,080 (31.6)	661,942 (93.0)
West Sumatra	–	78	68 (87.2)	111	1,382,872	0.80	283,735 (20.5)	1,336,770 (96.7)
South Sumatra	–	89	81 (91.0)	153	2,144,076	0.71	510,021 (23.8)	2,012,087 (93.8)
North Sumatra	1 (3.0)	212	184 (86.8)	284	3,767,771	0.75	1,237,200 (32.8)	3,604,383 (95.7)
West Papua	2 (28.6)	12	10 (83.3)	11	168,700	0.65	23,910 (14.2)	135,995 (80.6)
Southwest Papua	2 (33.3)	12	9 (75.0)	12	143,018	0.84	48,784 (34.1)	117,974 (82.5)
**INDONESIA**	**32 (6.2)**	**3,202**	**2,855 (89.2)**	**4,857**	**75,217,642**	**0.65**	**23,170,537 (30.8)**	**71,085,957 (94.5)**

HAPO = hospital with an actively practicing OBGYN.

### Maternal mortality rate analysis

**Figure 3:**
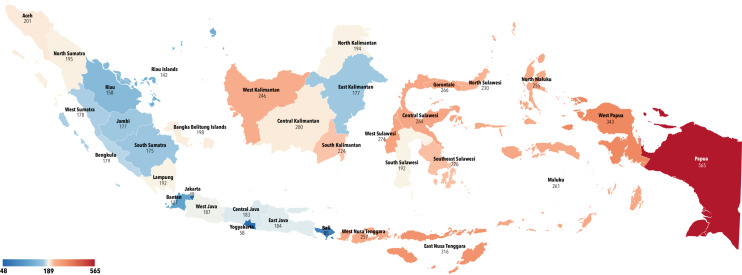
Maternal mortality ratio by provinces in 2020.

**Figure 4:**
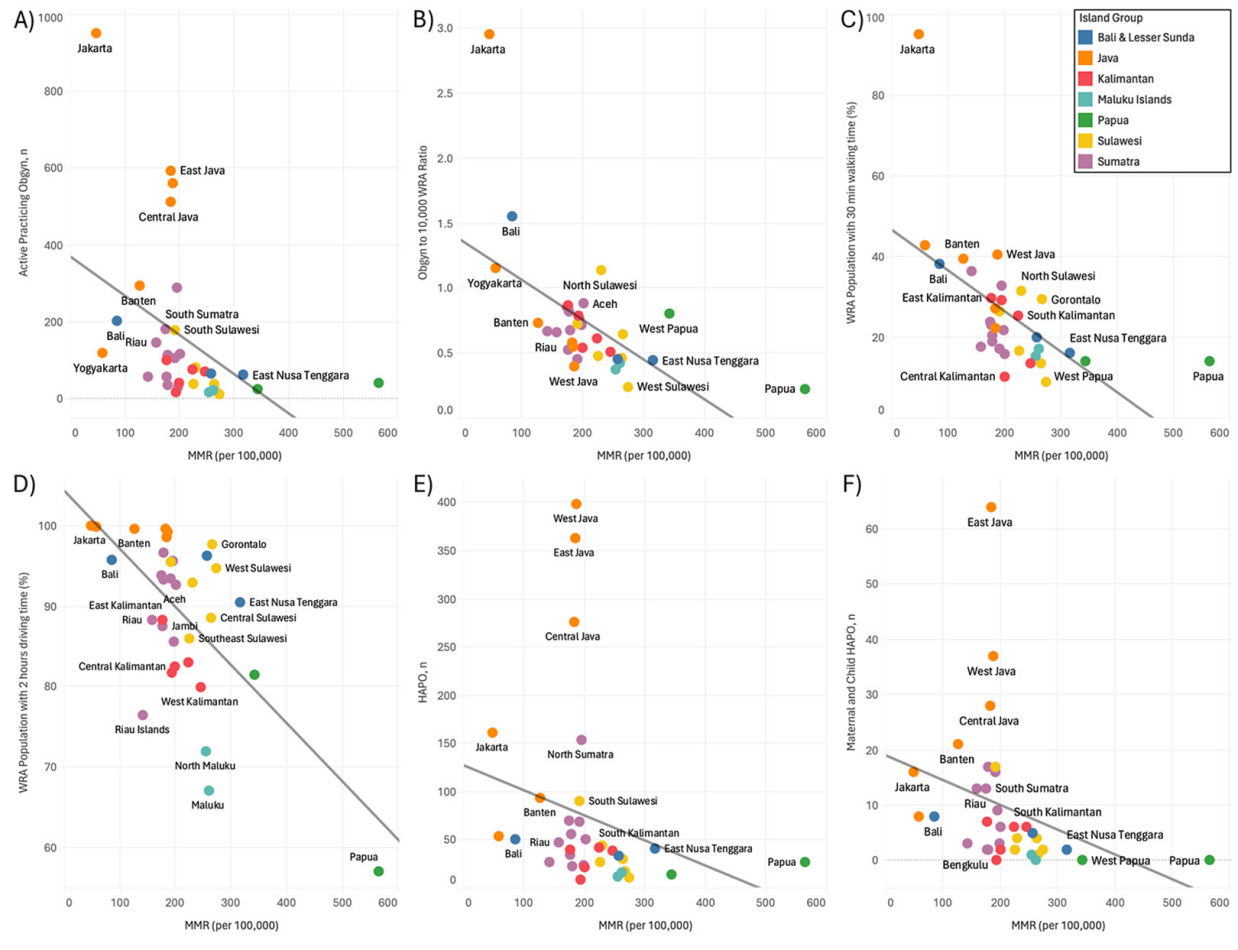
The MMR (per 100,000) was significantly and negatively correlated with the number of APOs (**A**), the ratio of OBGYN‑to‑10,000‑WRAs (**B**), the percentage of the WRA population within a 30‑min walking distance (**C**), and the percentage of the WRA population within 2HA (**D**). No significant correlation was observed between the MMR and the number of HAPOs (**E**) or MMR and the number of maternal and child HAPOs (**F**).

Indonesia’s national maternal mortality ratio (MMR) in 2020 was 186 per 100,000 live births. However, the MMR levels vary greatly in each province in Indonesia (Figure 3). As shown in Figure 4, the MMR (per 100,000) is significantly and negatively correlated with the number of APOs (*p* < 0.01, *R*² = 0.19; Figure 4A), the OBGYN‑to‑10,000‑WRA ratio (*p* < 0.001, *R²* = 0.33; Figure 4B), the percentage of the WRA population within a 30‑minute walking distance (*p* < 0.001, *R*² = 0.34; Figure 4C), and the percentage of the WRA population within 2HA (*p* < 0.0001, *R*² = 0.41; Figure 4D). However, no significant correlation was observed between the MMR and the number of hospitals with an actively practicing OBGYN (HAPOs; *p* = 0.16, *R*² = 0.06; Figure 4E), or between the MMR and the number of maternal and child HAPOs (*p* = 0.07, *R*² = 0.10; Figure 4F).

## Discussion

This is a novel study on the location and coverage of obstetric‑gynecological services in Indonesia nationally and in the provinces. In addition, it is the first study to utilize geospatial analysis in calculating reproductive‑age population estimates with access to emergency obstetric services in these provinces within a set time. This aligns with and adds to findings from similar studies in Southeast Asia, specifically in Malaysia and the Philippines, where the national 2HA target set by the LCoGS was achieved [5, 6]. However, regional disparities are evident in remote and underserved areas. These studies highlight that significant gaps in access persist locally, particularly in regions with challenging geography and lower population densities, despite averages meeting global standards.

Access to facilities with emergency obstetric services in Indonesia varies depending on geographic location. Based on region, there were evident disparities in access to essential surgery, as eight provinces did not meet the LCoGS target. This implies that health equity in surgery varies across the different regions of Indonesia. Moreover, despite having more than 4,857 active OBGYNs, only 2,855 of the 3,202 hospitals had OBGYNs. This implies that many OBGYNs have multisite practices in locations where other OBGYNs are present. Such clustering may lead to inaccuracies in assessing physician availability in specific areas, which can hinder the development of effective interventions and public policies, as observed in the United States [12].

Specialist doctors, particularly OBGYNs, usually establish their practices in densely populated areas, as previously reported in an Indonesian study [13]. This clustering can be attributed to multiple factors, including the assurance of better welfare, access to advanced medical facilities, professional growth opportunities, and higher patient volumes [14]. Urban areas often offer more robust healthcare infrastructure, better educational opportunities for children, and enhanced personal and professional networks, making these locations more attractive for specialists [15]. However, this concentration underscores the disparity in healthcare access between urban and rural areas, highlighting the need for targeted policies to distribute medical professionals evenly across regions.

Global surgery, which encompasses essential obstetric surgical services, plays a pivotal role in achieving the Sustainable Development Goals 2030 (SDGs) by addressing key objectives such as eradicating poverty (SDG 1), enhancing health and well‑being (SDG 3), promoting economic growth and decent work (SDG 8), and reducing inequalities (SDGs 5 and 10) [16]. This broad impact highlights the importance of accessible and comprehensive surgical, anesthetic, and obstetric (SAO) care, particularly in regions with geographical challenges limiting service availability.

Geographical access to SAO care must be clearly defined, as it can facilitate the expansion of surgical care and strategy development to enhance geographic access for the population. SAO care includes a wide array of procedures, with obstetric care forming a crucial foundation that significantly affects WRA. Numerous studies have evaluated geographical surgical access using GIS software in various countries [5, 6, 11, 17–23]. Any effort to scale up surgical care access requires a thorough evaluation of the distribution of facilities providing such care. The data gathered in this study should be integrated into Indonesia’s National Surgical, Obstetric, Anesthesia, and Nursing Plan (NSOANP), representing an initial critical step toward enhancing the surgical system and improving surgical care [24]. The geospatial analysis results can inform national surgical planning and policy development, aiming to improve access to safe, affordable, and timely surgical care.

According to geospatial analysis, eight provinces have failed to meet the first LCoGS indicator objective of 80%. The higher maternal mortality rate (MMR) levels in certain provinces—Papua province, for instance, had 565 per 100,000 live births—is consistent with this reality. This number is significantly higher than the national MMR (186 per 100,000 live births). These data contrast sharply with the MMR levels of 48 per 100,000 in Jakarta province, which has the highest 2HA‑to‑EOSC ratio [25].

The significant negative correlation between the maternal mortality ratio (MMR) and the number of active practicing obstetricians and gynecologists (APOs), as well as the OBGYN‑to‑10,000‑women‑of‑reproductive‑age (WRA) ratio, underscores the critical role of skilled obstetric care availability in reducing maternal deaths. This relationship suggests that provinces with a higher density of practicing obstetricians experience lower maternal mortality rates, likely due to improved access to timely and competent care during pregnancy and childbirth. This finding aligns with previous research indicating that the availability of human resources, such as obstetricians and midwives, is correlated with reduced insufficient referrals and better maternal outcomes [26].

Conversely, the absence of a significant correlation between MMR and the number of hospitals with an available OBGYN (HAO) suggests that merely increasing the number of such facilities does not necessarily lead to improved maternal health outcomes. This discrepancy may be attributed to factors such as the uneven distribution of obstetricians across hospitals, service quality, and readiness variations, as well as barriers to accessing these facilities, including geographic distance and socioeconomic constraints. Studies have highlighted that, despite the presence of healthcare facilities, disparities in access and quality of care persist, contributing to high maternal mortality rates [27]. Therefore, addressing maternal mortality requires a comprehensive approach that ensures equitable distribution of qualified healthcare providers and addresses barriers to accessing high‑quality maternal care.

Indonesia has achieved a national CS rate of 17.6%, using the 2018 Indonesian Demographic Health Survey [28]. This surpasses the World Health Organization’s recommended threshold of 10–15% for deliveries to reduce MMR levels [29]; however, the country still reports high MMR levels [25]. This discrepancy underscores the significant gap in maternal health outcomes. While factors, including proximity to health facilities and urban residency, contribute to higher CS rates [30], they do not fully address the complexities of maternal mortality. This indicates potential gaps in the healthcare system, such as variations in the quality of care, rural access problems, and underlying health conditions in pregnant women, which are not resolved by higher CS rates alone. A deeper evaluation of these factors is essential to understand the persistently high MMR despite meeting the CS targets, suggesting the need for more comprehensive maternal healthcare strategies.

The LCoGS identified three factors causing delays in patient care: (1) lack of knowledge about health systems, poor health‑seeking behavior, or distrust in the health system and cultural beliefs; (2) poor accessibility to healthcare facilities due to costs or infrastructure; and (3) insufficient capacity of health services to provide necessary care upon arrival [31]. The 2‑h bellwether access in our geospatial analysis study only addresses the travel aspect of the second delay and does not account for travel costs, including ambulance services, which are not free in Indonesia and often face logistical challenges along with long response times [32]. These travel costs and personal family needs can contribute to the first and second delays [32]. In addition, a late referral system can worsen delays, leading to maternal mortality as evidenced by previous study. Therefore, further studies should consider these other “delays” to improve access to SAO care [33].

Our study had some methodological limitations. First, the Indonesian archipelago, with over 17,000 islands and a total area of approximately 5.1 million km², is predominantly water‑logged (approximately 70%). Similar to a study conducted in the Philippines [6], we excluded boats and air transportation from our ArcGIS analysis. The variability in air and sea travel time influenced by the weather conditions and transportation type (from ferries to canoes), including challenging terrain in mountainous regions, such as Papua, where access may depend solely on helicopters or non‑commercial flights with infrequent schedules, made us standardize our measurements to land transportation times. Consequently, residents of remote islands requiring air or sea travel to reach healthcare facilities were categorized as outside the 2HA zone for hospitals with available OBGYNs. In this study, we considered other influential travel factors, such as economic, social, or cultural aspects.

There is a possibility that the 2HA estimations are optimistic because we did not account for variability in traffic conditions and temporary road closures due to seasonal climate change, monsoons, floods, or earthquakes, which are frequent in Indonesia, a tropical country located in the Ring of Fire. In addition, the road network dataset in Indonesia may not accurately represent travel conditions due to the exclusion of unnamed and smaller roads. Furthermore, population datasets may either under‑ or overestimate the population of certain areas, leading to distorted results.

Moreover, not all Indonesians can afford cars or other motorized vehicles, which may result in variations in the calculated 2HA estimates. According to the Indonesian Central Bureau of Statistics, the number of registered cars and motorcycles in 2022 was 17,168,862 and 125,305,332, respectively. This translates to an estimated six cars and 45 motorcycles per 100 persons. Despite these figures being relatively low, there are multiple alternatives and affordable modes of public transportation tailored to local customs in various regions, including *angkot* (minivans), *bajaj* (three‑wheeled taxis), *mikrolet* (minibuses), *becak* (pedicabs), *bentor* (motorized pedicabs), *bendi* (horse‑drawn carriages), and *oplet* (vans). In addition, online motorcycle and car taxi services, such as Gojek, are available in almost all major cities and regencies in Indonesia and can be booked 24/7, depending on driver availability.

In this study, we assumed that hospitals can provide emergency obstetric surgical procedures 24/7. However, some facilities may not continue to operate, and the availability of OBGYNs and functional operating theaters is limited in certain regions. The surgical safety checklist for cesarean sections developed by the Society for Maternal‑Fetal Medicine (SMFM) may not be followed by all hospitals in Indonesia [34]. Furthermore, the shortage of anesthesiologists in some areas may result in surgeries being performed without adequate anesthesia, which is suboptimal and can lead to undesirable outcomes [35].

Owing to technical and logistical constraints, we were unable to conduct a facility assessment across all 2,566 of the 3,117 hospitals in Indonesia. We recommend that future studies should include such assessments because data discrepancies are significant problems in Indonesia and are simultaneously being addressed and improved through the One Data Indonesia initiative by the government [36].

## Conclusion

In conclusion, 94.5% of the Indonesian WRA populations were able to reach a hospital with EOSC within 2 h. However, the provincial analysis showed that access is below the LCoGS’s recommendation of 80% in the eight provinces. The results of this study will help inform the Indonesian government when it comes to national obstetric planning.

## Verification

All authors had access to the data.

**BAT:** Project leader, conceptualization, data curation, methodology, validation, funding acquisition, and writing—original draft.

**HAS:** Conceptualization, data curation, formal analysis, methodology, software, validation, funding acquisition, and writing—original draft.

**FRM:** Conceptualization, data curation, methodology, validation, funding acquisition, and writing—original draft.

**RAU:** Data curation, validation, writing—original draft, and writing—review and editing.

**AI:** Data curation, validation, funding acquisition, and writing—review and editing.

**BP:** Data curation, validation, and writing—review and editing.

**KN**, **BW, PHH, AAIL**, and **MMHA:** Writing—review and editing.

## Additional File

The additional file for this article can be found as follows:

Supplementary File: Figure 1. Stratification of Indonesian hospitals by class.

Supplementary File: Table 1. Characteristic of OBGYN practice sites.

Supplementary File: Table 2. Type of hospital without an actively practicing OBGYN.

Supplementary File: Table 3. Class stratification of hospital without an actively practicing OBGYN.
