# Supplementary figures and images for: Geospatial Access to Emergency Obstetric Surgery in Indonesia: Is Travel Time for Access Too Long?
Source: Ann Glob Health. 2024 Dec 28;90(1):82. doi: 10.5334/aogh.4598 (PMC11697619; doi:10.5334/aogh.4598)

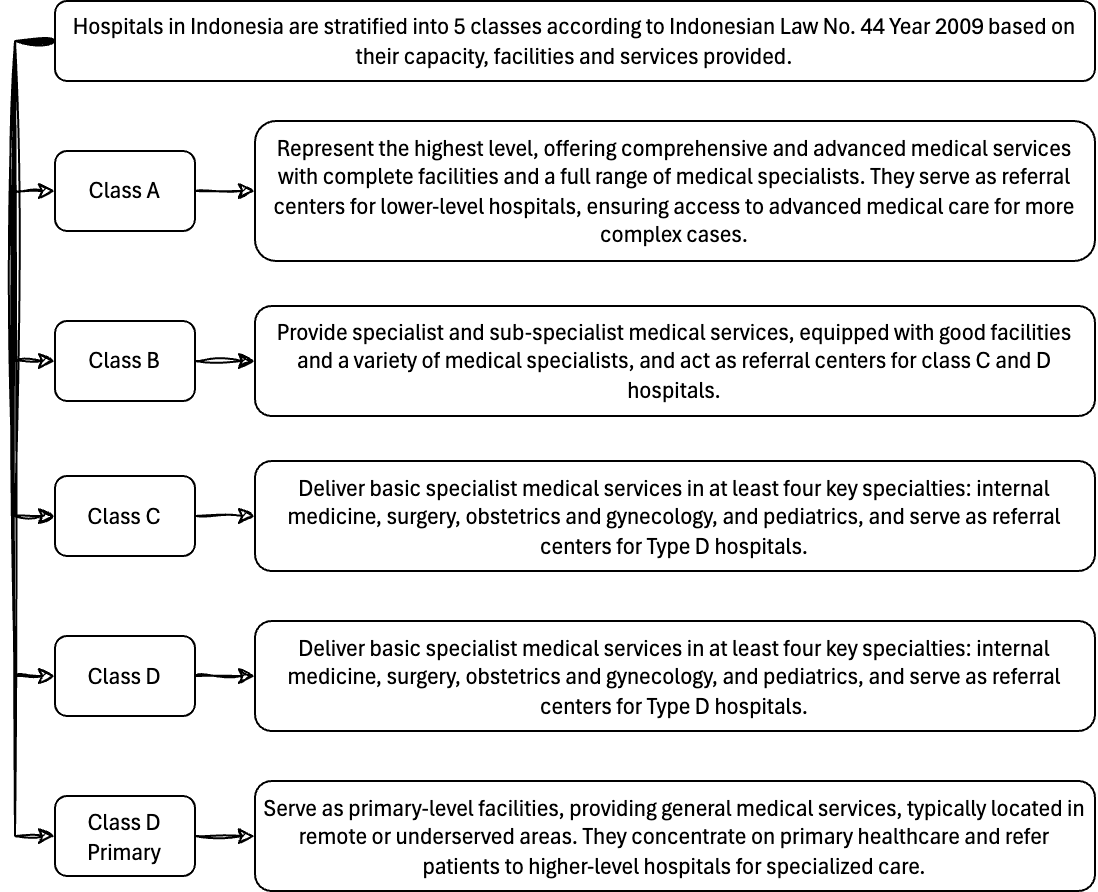

Supplement: Supplementary File: Figure 1. — Stratification of Indonesian hospitals by class. [file agh-90-1-4598-s1.png]
