# Supplementary material for: Geospatial Access to Emergency Obstetric Surgery in Indonesia: Is Travel Time for Access Too Long?
Source: Ann Glob Health. 2024 Dec 28;90(1):82. doi: 10.5334/aogh.4598 (PMC11697619; doi:10.5334/aogh.4598)
Supplement: Supplementary File: Table 1. — Characteristic of OBGYN practice sites. [file agh-90-1-4598-s2.pdf]

Supplementary Table 1. Characteristic of OBGYN Practice Sites

**Province**	**Total OBGYN**	**Inactive/Retired OBGYN, n (%)**	**OBGYN in Private Clinic Only, n (%)**
Aceh	119	14 (11.8)	3 (2.5)
Bali	203	10 (4.9)	4 (2.0)
Banten	294	52 (17.7)	12 (4.1)
Bengkulu	34	2 (5.9)	-
Jakarta	957	158 (16.5)	45 (4.7)
Yogyakarta	123	25 (20.3)	3 (2.4)
Gorontalo	22	-	-
Jambi	57	1 (1.8)	-
West Java	566	13 (2.3)	8 (1.4)
Central Java	512	22 (4.3)	10 (2.0)
East Java	593	9 (1.5)	7 (1.2)
West Kalimantan	69	2 (2.9)	1 (1.4)
South Kalimantan	77	4 (5.2)	2 (2.6)
Central Kalimantan	40	1 (2.5)	-
East Kalimantan	101	7 (6.9)	5 (5.0)
North Kalimantan	17	1 (5.9)	-
Bangka Belitung Islands	31	-	2 (6.5)
Riau Islands	57	17 (29.8)	-
Lampung	105	9 (8.6)	1 (1.0)
Maluku	23	1 (4.3)	1 (4.3)
North Maluku	15	1 (6.7)	-
West Nusa Tenggara	66	2 (3.0)	3 (4.5)
East Nusa Tenggara	61	-	-
Papua	21	-	-
Highland Papua	5	-	-
South Papua	6	-	-
Central Papua	8	-	-
Riau	146	31 (21.2)	5 (3.4)
West Sulawesi	11	1 (9.1)	-
South Sulawesi	185	8 (4.3)	1 (0.5)
Central Sulawesi	39	-	-
Southeast Sulawesi	37	-	-
North Sulawesi	82	5 (6.1)	2 (2.4)
West Sumatra	113	2 (1.8)	1 (0.9)
South Sumatra	182	29 (15.9)	1 (0.5)
North Sumatra	303	19 (6.3)	3 (1.0)
West Papua	13	2 (15.4)	-
Southwest Papua	12	-	-
**INDONESIA**	**5305**	**448 (8.4)**	**120 (2.3)**
