# Supplementary material for: Geospatial Access to Emergency Obstetric Surgery in Indonesia: Is Travel Time for Access Too Long?
Source: Ann Glob Health. 2024 Dec 28;90(1):82. doi: 10.5334/aogh.4598 (PMC11697619; doi:10.5334/aogh.4598)
Supplement: Supplementary File: Table 2. — Type of hospital without an actively practicing OBGYN. [file agh-90-1-4598-s3.pdf]

Supplementary Table 2. Type of Hospital without Active Practicing OBGYN

Province	General Hospital		Maternal and Child Hospital		Surgical Hospital		Other Category Hospital	
	n	(%)	n	(%)	n	(%)	n	(%)
Aceh	8	11.3	-	-	1	100.0	2	100.0
Bali	5	7.6	-	-	1	33.3	4	100.0
Banten	1	1.0	-	-	1	100.0	3	100.0
Bengkulu	1	4.3	-	-	-	-	1	100.0
Jakarta	-	-	-	-	2	40.0	25	83.3
Yogyakarta	4	6.3	-	-	3	75.0	6	100.0
Gorontalo	2	10.5	-	-	-	-	-	-
Jambi	3	7.7	-	-	-	-	1	100.0
West Java	1	0.3	-	-	2	100.0	15	78.9
Central Java	7	2.3	-	-	1	33.3	13	76.5
East Java	4	1.1	-	-	4	80.0	13	81.3
West Kalimantan	8	16.0	-	-	-	-	2	100.0
South Kalimantan	1	2.4	-	-	-	-	2	100.0
Central Kalimantan	5	16.7	-	-	-	-	1	100.0
East Kalimantan	8	16.0	-	-	-	-	3	100.0
North Kalimantan	7	41.2	-	-	-	-	-	-
Bangka Belitung Islands	2	8.3	-	-	-	-	1	100.0
Riau Islands	4	12.5	-	-	-	-	0	0.0
Lampung	-	-	-	-	1	100.0	3	100.0
Maluku	9	31.0	-	-	-	-	1	100.0
North Maluku	4	19.0	-	-	-	-	1	100.0
West Nusa Tenggara	2	5.1	-	-	-	-	2	100.0
East Nusa Tenggara	16	26.2	-	-	-	-	1	100.0
Papua	3	17.6	-	-	-	-	1	100.0
Highland Papua	7	77.8	-	-	-	-	0	-
South Papua	1	12.5	-	-	-	-	0	-
Central Papua	8	57.1	-	-	-	-	0	-
Riau	7	10.6	-	-	-	-	3	100.0
West Sulawesi	6	42.9	-	-	-	-	0	-
South Sulawesi	11	11.2	1	5.6	-	-	6	75.0
Central Sulawesi	9	25.0	-	-	-	-	0	-
Southeast Sulawesi	3	8.6	-	-	-	-	2	100.0
North Sulawesi	5	10.2	-	-	-	-	5	100.0
West Sumatra	2	3.9	-	-	2	100.0	6	75.0
South Sumatra	5	6.9	-	-	1	100.0	2	66.7
North Sumatra	15	8.0	-	-	1	50.0	12	92.3
West Papua	2	16.7	-	-	-	-	0	-
Southwest Papua	3	25.0	-	-	-	-	0	-
**INDONESIA**	**189**	**7.0**	**1**	**0.3**	**20**	**64.5**	**137**	**85.6**
