# Supplementary material for: Geospatial Access to Emergency Obstetric Surgery in Indonesia: Is Travel Time for Access Too Long?
Source: Ann Glob Health. 2024 Dec 28;90(1):82. doi: 10.5334/aogh.4598 (PMC11697619; doi:10.5334/aogh.4598)
Supplement: Supplementary File: Table 3. — Class stratification of hospital without an actively practicing OBGYN. [file agh-90-1-4598-s4.pdf]

Supplementary Table 3. Class Stratification of Hospital without Active Practicing OBGYN

Province	Class A	Class B	Class C	Class D	Class D Primary	Class Not Defined
Aceh	1 (50.0)	1 (7.7)	2 (6.1)	6 (19.4)	1 (100.0)	-
Bali	2 (66.7)	1 (8.3)	5 (9.6)	1 (7.7)	1 (100.0)	-
Banten	-	-	4 (4.7)	1 (5.0)	-	-
Bengkulu	-	1 (50.0)	-	1 (12.5)	-	-
Jakarta	6 (31.6)	8 (13.3)	13 (15.3)	-	-	-
Yogyakarta	2 (66.7)	2 (14.3)	6 (21.4)	3 (8.3)	-	-
Gorontalo	-	-	-	-	2 (100.0)	-
Jambi	-	1 (25.0)	-	2 (15.4)	-	1 (100.0)
West Java	4 (44.4)	6 (7.5)	7 (2.7)	1 (1.2)	-	-
Central Java	3 (27.3)	2 (5.9)	9 (4.7)	7 (5.9)	-	-
East Java	2 (28.6)	4 (6.3)	12 (5.4)	3 (2.1)	-	-
West Kalimantan	-	1 (33.3)	1 (3.1)	5 (26.3)	3 (100.0)	-
South Kalimantan	1 (50.0)	1 (14.3)	-	1 (6.7)	-	-
Central Kalimantan	-	1 (25.0)	-	-	5 (100.0)	-
East Kalimantan	1 (33.3)	-	2 (6.9)	6 (30.0)	2 (66.7)	-
North Kalimantan	-	-	-	2 (40.0)	5 (83.3)	-
Bangka Belitung Islands	-	1 (50.0)	-	1 (12.5)	1 (50.0)	-
Riau Islands	-	-	1 (5.3)	3 (37.5)	-	-
Lampung	-	1 (25.0)	3 (5.3)	-	-	-
Maluku	-	1 (33.3)	1 (10.0)	6 (40.0)	2 (100.0)	-
North Maluku	-	-	1 (16.7)	3 (23.1)	1 (50.0)	-
West Nusa Tenggara	-	1 (25.0)	1 (4.3)	1 (5.9)	1 (100.0)	-
East Nusa Tenggara	-	-	2 (6.3)	7 (36.8)	8 (80.0)	-
Papua	-	1 (50.0)	-	1 (25.0)	2 (100.0)	-
Highland Papua	-	-	-	4 (80.0)	2 (100.0)	1 (100.0)
South Papua	-	-	-	-	1 (100.0)	-
Central Papua	-	-	-	5 (71.4)	2 (100.0)	1 (100.0)
Riau	1 (50.0)	-	5 (10.6)	4 (14.8)	-	-
West Sulawesi	-	-	-	4 (80.0)	2 (100.0)	-
South Sulawesi	1 (33.3)	2 (7.1)	8 (11.6)	1 (5.9)	6 (85.7)	-
Central Sulawesi	-	-	1 (4.3)	4 (50.0)	4 (80.0)	-
Southeast Sulawesi	-	2 (50.0)	1 (5.9)	2 (11.1)	-	-
North Sulawesi	-	2 (33.3)	3 (8.3)	5 (33.3)	-	-
West Sumatra	-	3 (42.9)	5 (9.8)	2 (13.3)	-	-
South Sumatra	-	1 (14.3)	2 (3.6)	3 (13.6)	2 (66.7)	-
North Sumatra	-	2 (7.4)	13 (10.7)	11 (18.6)	2 (100.0)	-
West Papua	-	-	1 (14.3)	1 (20.0)	-	-
Southwest Papua	-	-	-	1 (14.3)	2 (100.0)	-
**INDONESIA**	**24 (31.6)**	**46 (10.3)**	**109 (6.3)**	**108 (12.3)**	**57 (82.6)**	**3 (100.0)**
